# ARID1A deficiency reverses the response to anti-PD(L)1 therapy in *EGFR-*mutant lung adenocarcinoma by enhancing autophagy-inhibited type I interferon production

**DOI:** 10.1186/s12964-022-00958-5

**Published:** 2022-10-13

**Authors:** Dantong Sun, Haili Qian, Jinsong Wang, Tongji Xie, Fei Teng, Junling Li, Puyuan Xing

**Affiliations:** 1 Department of Medical Oncology, National Cancer Center/National Clinical Research Center for Cancer/Cancer Hospital, Chinese Academy of Medical Sciences and Peking Union Medical College, Beijing, 100021 China; 2 State Key Laboratory of Molecular Oncology, National Cancer Center/National Clinical Research Center for Cancer/Cancer Hospital, Chinese Academy of Medical Sciences and Peking Union Medical College, Beijing, 100021 China

**Keywords:** *EGFR*-mutant lung adenocarcinoma, ARID1A, Immunotherapy, Autophagy, Type I interferon

## Abstract

**Introduction:**

*EGFR* mutations in non-small cell lung cancer (NSCLC) are associated with a poor response to immune checkpoint inhibitors (ICIs), and only 20% of NSCLC patients harboring *EGFR* mutations benefit from immunotherapy. Novel biomarkers or therapeutics are needed to predict NSCLC prognosis and enhance the efficacy of ICIs in NSCLC patients harboring *EGFR* mutations, especially lung adenocarcinoma (LUAD) patients, who account for approximately 40–50% of all NSCLC cases.

**Methods:**

An ARID1A-knockdown (ARID1A-KD) *EGFR*-mutant LUAD cell line was constructed using lentivirus. RNA-seq and mass spectrometry were performed. Western blotting and IHC were used for protein expression evaluation. Effects of 3-MA and rapamycin on cells were explored. Immunofluorescence assays were used for immune cell infiltration examination.

**Results:**

ARID1A expression was negatively associated with immune cell infiltration and immune scores for ICIs in LUAD with *EGFR* mutations. In vitro experiments suggested that ARID1A-KD activates the EGFR/PI3K/Akt/mTOR pathway and inhibits autophagy, which attenuates the inhibition of Rig-I-like receptor pathway activity and type I interferon production in *EGFR*-mutant LUAD cells. In addition, 3-MA upregulated production of type I interferon in *EGFR*-mutant LUAD cells, with an similar effect to ARID1A-KD. On the other hand, rapamycin attenuated the enhanced production of type I interferon in ARID1A-KD *EGFR*-mutant LUAD cells. ARID1A function appears to influence the tumor immune microenvironment and response to ICIs.

**Conclusion:**

ARID1A deficiency reverses response to ICIs in *EGFR*-mutant LUAD by enhancing autophagy-inhibited type I interferon production.

**Video Abstract**

**Supplementary Information:**

The online version contains supplementary material available at 10.1186/s12964-022-00958-5.

## Introduction

Lung cancer ranks first in mortality among all malignancies worldwide, and it has become a serious public health problem. Lung adenocarcinoma (LUAD) is one of the most common pathological types of non-small cell lung cancer (NSCLC) and accounts for approximately 40–50% of all Asian NSCLC cases [1, 2]. Approximately 40% of Asian LUAD patients harbor mutations in driver genes and might benefit from targeted therapy, such as tyrosine kinase inhibitors (TKIs) targeting sensitive mutations in epidermal growth factor receptor (EGFR) [3] or anaplastic lymphoma kinase (ALK) [4]. Although mutations in exons 18–21 of the *EGFR* gene have been detected, the majority of *EGFR* mutations comprise exon 19 deletions and exon 21 substitutions of leucine for arginine (L858R) [5, 6]. LUAD patients harboring sensitive *EGFR* mutations can benefit from EGFR-TKI therapy, most develop resistance to the drugs and require further treatment.

Immune checkpoint inhibitors (ICIs), as represented by anti-programmed cell death (ligand) 1 [anti-PD(L)1] therapy, have been widely administered as the standard therapy for NSCLC without driver gene mutation. Multiple clinical trials have disclosed the important role of ICIs in benefiting the overall survival (OS) of patients [7–11]. However, only 20% of NSCLC patients harboring *EGFR* mutations respond to second-line immunotherapy treatment [12], leading to a dilemma in devising treatment strategies. Therefore, exploration of novel biomarkers to predict response to ICIs or to find new strategies to overcome insensitivity to ICI treatment in NSCLC with *EGFR* mutation is urgently needed.

Switch/sucrose nonfermenting (SWI/SNF) chromatin remodeling complexes maintain a series of biological processes related to cell development and differentiation [13, 14]. SWI/SNF family members are frequently dysregulated in various malignancies, leading to functional loss of the complexes [15]. The canonical BRG1/BRM-associated factor (BAF) complex, which is one of the three main final forms of assembled SWI/SNF chromatin remodeling complexes [16], mainly consists of ARID1A, ARID1B and DPF2 [17, 18]. In particular, ARID1A, which has biological functions as an essential molecule in DNA repair and stabilization [19], can be dysregulated in malignancies and is associated with cancer development. Previous research has revealed the function of *ARID1A* mutation in impairing mismatch repair (MMR) function, increasing the tumor mutation burden (TMB) and predicting good prognosis to cancer immunotherapy in vivo [20]. The majority of *ARID1A* mutations are inactivating mutations and result in loss of ARID1A expression [20]; therefore, ARID1A expression loss should also be associated with response to immunotherapy. In addition, whether the role of *ARID1A* mutation or expression loss has the same effect in immunotherapy among *EGFR*-mutant LUAD patients remains to be elucidated. In this study, we found that ARID1A expression was negatively associated with tumor-infiltrating lymphocytes (TILs) and immune scores for ICIs. Furthermore, in vitro experiments suggested that ARID1A knockdown (ARID1A-KD) activates the EGFR/PI3K/Akt/mTOR pathway and inhibits tumor cell autophagy, which attenuates inhibition of the Rig-I-like receptor pathway and production of type I interferon (IFN) in *EGFR-*mutant LUAD cells. In summary, ARID1A influences the tumor immune microenvironment (TIME) and response to ICIs.

## Materials and methods

### Bioinformatic analyses

Pan-cancer datasets from the cBioPortal for Cancer Genomics (the cBioPortal) [21, 22] were used to explore the effect of *ARID1A* mutation on the TMB and value of immunotherapy. A LUAD dataset is available at The Cancer Genome Atlas (TCGA) (http://cancergenome.nih.gov/). Immune estimation, including the immune score, stromal score and ESTIMATE score [23], was used to clarify immune cell infiltration in LUAD. In addition, evaluation of the immunophenoscore (IPS) [24] was used to predict response to ICI treatment. In this study, the gene set of pancancer immune cells derived from a published study [24] was employed to estimate immune cell infiltration via the single-sample Gene Set Enrichment Analysis (ssGSEA) method, and infiltrating immune cells were clarified and compared between LUAD patients subgrouped by ARID1A expression (best cutoff: 75% percentile of ARID1A expression). To explore functions or pathways associated with differentially expressed genes (DEGs) according to ARID1A expression in LUAD patients from TCGA, Gene Set Enrichment Analysis (GSEA) [25] was utilized via GSEA 4.0.3. The analysis was performed using the GOBP database.

### Patients

In total, 53 LUAD patients harboring sensitive *EGFR* mutations (19del or 21L858R) who were admitted to our cancer center between August 2012 and September 2021 were enrolled in this study. The basic characteristics of these patients, including age, sex, *EGFR* mutation type, T790M status and treatment information, were collected, as listed in Table 1. All patients were diagnosed with stage IV LUAD in our pathology department after the evaluation of hematoxylin and eosin (H&E) staining and immunohistochemistry (IHC); the last follow-up time was March 2022. All patients had received first-line and/or second-line EGFR-TKI treatment, with 10 patients in the cohort receiving subsequent ICI treatment (combined with chemotherapy and/or antivascular therapy) after EGFR-TKI failure. The disease status of each patient was evaluated using the standard of Response Evaluation Criteria in Solid Tumors 1.1 (RECIST 1.1), and all tumors were staged according to the 2019 American Joint Committee on Cancer (AJCC) TNM staging system for lung cancer [26]. The Ethics Committee of Cancer Hospital Chinese Academy of Medical Sciences approved this study (No. NCC-007421), and all investigations were carried out according to the rules of the Declaration of Helsinki. All experiments were conducted following National Health and Family Planning Commission of the Professional Regulation Commission (PRC) guidelines.

**Table 1:** Information for the *EGFR*-mutant LUAD patients involved in this study

Variables	Patient number (n)
Sex	
Male	26
Female	27
*EGFR* mutation types	
Exon 19 deletion	24
Exon 21 L858R	29
T790M status	
Positive	32
Negative	21
Age	
≤ 60 years	29
> 60 years	24
ECOG	
0–1	44
2	9
Treatment information	
First line	
1st generation EGFR-TKIs	43
3rd generation EGFR-TKIs	10
Second line	
3rd generation EGFR-TKIs	40
ARID1A expression	
High expression	31
Low expression	22

### IHC assay for ARID1A expression

IHC slides to evaluate ARID1A expression were collected after obtaining informed consent from the enrolled patients. Five-micrometer-thick sections were cut from paraffin-embedded tissues for IHC examination. Antigen retrieval was performed by boiling the slides in 10 mM citrate buffer (pH 6.0) for 10 min, followed by cooling at room temperature for 20 min. Each section was incubated with primary antibodies against ARID1A at appropriate concentrations (1:500) overnight at 4 °C. Two investigators independently evaluated the IHC slides, and five fields of each slide were selected for the evaluation of IHC scores. We followed a previously reported scoring method [27]. The intensity of staining was scored as 0 (no staining), 1 (weak), 2 (medium) or 3 (strong). Percent scores were assigned as 0 (< 5%), 1 (5–25%), 2 (26–50%), 3 (51–75%) and 4 (76–100%). The final score of each slide was calculated as the average score of the 5 fields selected randomly and ranged from 0 to 12 (intensity score x percentage score). Specifically, low expression of ARID1A was defined as a final score less than 9 (IHC score < 9, 75% percentile of ARID1A IHC score, according to the best cutoff value revealed by bioinformatic analysis). Information about the antibodies used is listed in Additional file 1: Table S1. We used human kidney tissue as a positive control for ARID1A IHC analyses and normal human lung tissue as a negative control.

### Cell lines and construction of stable infectants [27]

The HCC4006 LUAD cell line with a sensitive *EGFR* mutation (ATCC No.: CRL-2871) was purchased from the Cell Bank of the Chinese Academy of Sciences (Shanghai, China). Information for the STR Cell ID assay is provided in Additional file 3: File 1. In addition, RNA samples of ARID1A-KD and NC cells derived from the A549 cell line (kindly supplied by Prof. Helei Hou, from the Affiliated Hospital of Qingdao University), an *EGFR* wild-type human LUAD cell line, were used to perform subsequent analysis to verify the findings. Cells were cultured in RPMI-1640 medium supplemented with 10% fetal bovine serum (FBS) and 1% P/S (100 IU/ml penicillin and 100 IU/ml streptomycin) in a 37 °C humidified atmosphere with 5% CO_2_. Lentiviral vectors encoding short hairpin RNAs (shRNAs) for ARID1A and a corresponding vector control (negative control [NC]) were purchased from GeneChem (Shanghai, China). Using the helper solution (GeneChem, Shanghai, China), cells were infected with lentiviruses according to the manufacturer’s instructions, after which the infection efficiency was verified by fluorescence microscopy. Cell counting revealed that over 90% of cells expressed fluorescent protein as a preliminary evaluation of the infection efficiency, which was considered an appropriate efficiency. Subsequently, ARID1A expression was examined using western blotting. Stably infected cell strains were selected for seven days and cultured with 2 μg/ml puromycin (Solarbio, Beijing, China) [27]. The sh-ARID1A and the vector control sequences are listed in Additional file 1: Table S1.

### RNA-seq library construction and data analysis

TRIzol (Invitrogen, Carlsbad, CA) was used to extract total RNA from cultured cells. The total RNA was then treated with RQ1 DNase (Promega) to remove DNA. The quality and quantity of the purified RNA were determined by measuring absorbance at 260 nm/280 nm (A260/A280) using a SmartSpec Plus (Bio-Rad). For each sample, 5 μg of total RNA was used for RNA-seq library preparation. An Agilent 2100 bioanalyzer was employed for RNA quality assessment. NEBNext® Ultra™ Directional RNA Library Prep Kit for Illumina® was used for strand-specific library building, with Illumina sequencing by the synthesis method. The libraries were sequenced using an Illumina NovaSeq 6000 following the manufacturer’s instructions by Novogene Co., Ltd. (Beijing, China). Uniquely mapped reads were obtained to calculate the read number and FPKM (fragments per kilobase and per million) values for each gene. We used R studio software to analyze DEGs. A log2 (fold change) ≥ 1 and adjusted *P* ≤ 0.05 were set as thresholds to define DEGs [28].

### Mass spectrometry (MS) for total cellular protein

The label-free quantitative proteomics method was used for MS by Shanghai Bioprofile Technology Co., Ltd. Cell pellets were harvested, and total protein was extracted using SDT cell lysis reagent. Digested peptides were desalted using peptide desalting spin columns and lyophilized under vacuum. Peptide concentrations were measured using a Nanodrop. When performing the MS for phosphorylated proteins, the peptide solution was lyophilized under vacuum, and phosphorylated peptides were enriched with an Fe-NTA Phosphopeptide Enrichment Kit (Thermo, A32992); enriched phosphorylated peptides were collected according to the kit procedure for mass spectrometry analysis. An appropriate amount of enriched peptides for each sample was separated using a nanoliter flow rate Easy nLC 1200 chromatographic system (Thermo Scientific). MSFragger 3.4 software was used to retrieve data from UniProt Protein Data Bank (UniProt *Homo sapiens* (Human) [9606]-203800-202201.fasta). After comparison, a log2 (fold change) ≥ 1.5 (total proteins) or 2 (phosphorylated proteins) and *P* ≤ 0.05 were considered to indicate significantly different expression of modifier sites.

### Western blot (WB) analysis [27]

Whole protein lysates of cells scraped from culture dishes were collected using NP40 cell lysis reagent containing proteinase and phosphatase inhibitors (Beyotime) on ice for 30 min. Next, the cell lysates were centrifuged at 12,000 × *g* for 15 min at 4 °C, and the protein concentrations of the supernatants were determined using the BRADFORD method (Thermo Fisher). The supernatants were subsequently mixed with the corresponding volume of 5 × SDS loading buffer and heated at 100 °C for 10 min. Total protein (20 mg) of each sample was separated by SDS‒PAGE and transferred to 0.22 µm nitrocellulose membranes. The nitrocellulose membranes were blocked with 5% nonfat dry milk dissolved in PBST and incubated overnight with primary antibodies at appropriate dilutions (Additional file 1: Table S1). After washing with PBST solution three times for a total of 30 min, the nitrocellulose membranes were incubated with HRP-conjugated secondary antibodies on a shaker for 1 h at room temperature. An ECL reagent (Pierce, Rockford, IL, USA) was used to visualize the results. ImageJ software was used to evaluate target protein expression [27].

### Immunofluorescence assays (IFA)

Formaldehyde-fixed and paraffin-embedded sections from 49 *EGFR*-mutant LUAD patients were used for IFA. DAPI staining (blue) was utilized to label nuclei in sections, and IFA signals (arbitrary units [AU]) for CD3 (pink) and CD8 (green) were used for the evaluation of immune cell infiltration, representing total T lymphocytes and cytotoxic lymphocytes (CTLs), respectively. ImageJ software was applied to weigh the AU of IFA. Antibody information for IFA is listed in Additional file 1: Table S1.

### Statistical analyses

Nonparametric tests were used; *P* values were determined by two-tailed tests, and *P* < 0.05 was used to define statistical significance (*P* < 0.05: *; *P* < 0.01: **; *P* < 0.001: ***; *P* < 0.0001: ****) with GraphPad Prism 9.0 software (GraphPad, La Jolla, CA, USA). GOBP, KEGG and REACTOM databases were used for enrichment analyses based on DEGs according to RNA-seq or differentially expressed proteins (DEPs) according to MS. GraphPad Prism 9.0 software (GraphPad, La Jolla, CA, USA) and Bioinfo Intelligent Cloud (BIC) [29] were used for image plotting.

## Results

### ARID1A deficiency changes the immune phenotype and enhances immune cell infiltration in LUAD patients with or without EGFR mutations

**Fig. 1:**
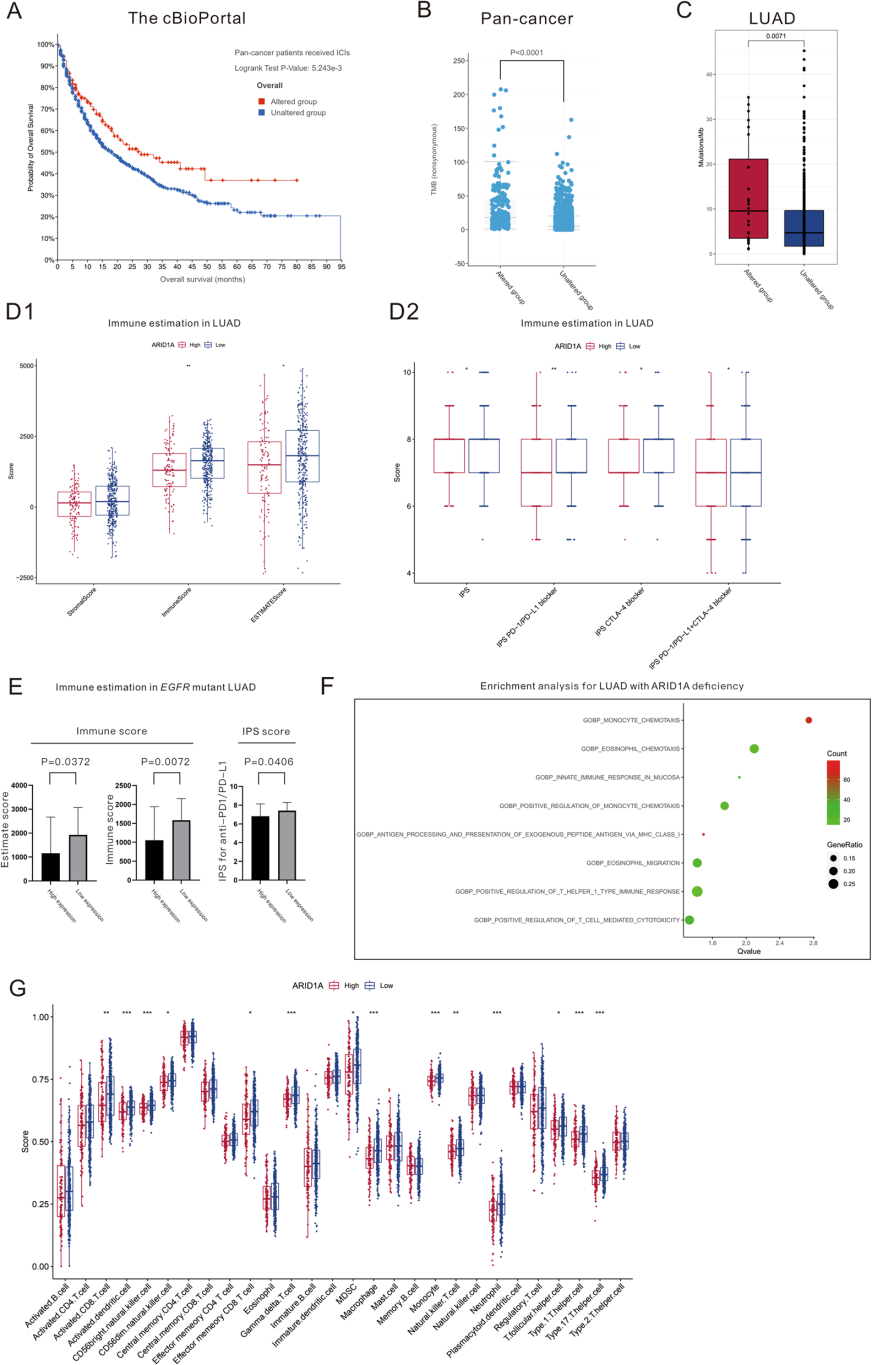
ARID1A deficiency changes the immune phenotype and enhances immune cell infiltration in lung adenocarcinoma patients with or without *EGFR* mutations. **A** *ARID1A* mutations were associated with longer overall survival in pancancer patients who received immunotherapy. **B** *ARID1A* mutations were associated with higher TMB values in pancancer patients. **C** *ARID1A* mutations were associated with higher TMB values in lung adenocarcinoma patients; **D1**, **D2** Low ARID1A expression was associated with higher ESTIMATE scores, immune scores and IPS scores in lung adenocarcinoma patients. **E** Low ARID1A expression was associated with higher ESTIMATE scores, immune scores and IPS scores (anti-PD1/PD-L1) in lung adenocarcinoma patients harboring *EGFR* mutation. **F** Enrichment analysis for lung adenocarcinoma patients with low ARID1A expression. **G** Lung adenocarcinoma patients with low ARID1A expression had a higher infiltrating immune cell abundance

Pan-cancer analysis (N = 1904) revealed that patients harboring *ARID1A* mutation (N = 291) had a longer OS than those with a wild-type gene after ICI administration (28.0 months versus 18.0 months, *P* = 0.0052), as displayed in Fig. 1A. *ARID1A* mutation was found to be related to the genomic instability of malignancy represented by a higher TMB both in pancancer patients (18.14/Mb versus 5.27/Mb, *P* < 0.0001, Fig. 1B) and LUAD patients (13.03/Mb versus 7.14/Mb, *P* = 0.0071, Fig. 1C). We then explored the role of ARID1A expression, which may be downregulated by *ARID1A* mutations, in cancer immunity and immunotherapy. Low ARID1A expression was related to the immunotherapy-sensitive phenotype in LUAD patients (N = 461), as characterized by higher ESTIMATE scores, immune scores (Fig. 1D1), and IPS scores for single agents of PD-1/PDL1 or CTLA-4 inhibitors or combined therapy (Fig. 1D2). Specifically, for LUAD patients harboring *EGFR* mutation (N = 64), low ARID1A expression still predicted a favorable response to immunotherapy, as represented by a higher ESTIMATE score (*P* = 0.0372), immune score (*P* = 0.0072) and IPS score for PD-1/PD-L1 inhibitors (*P* = 0.0406), as shown in Fig. 1E.

Mechanistically, enrichment analysis demonstrated that low ARID1A expression activates a variety of biological processes related to immunity, including antigen processing, immune cell chemotaxis and T-cell-mediated cytotoxicity upregulation (Fig. 1F). ssGSEA was performed to compare different statuses of immune cell infiltration between different groups of LUAD patients divided by ARID1A expression, as shown in Fig. 1G. The abundance of the majority of infiltrating immune cells was higher in the ARID1A low expression group, revealing a phenotype of abundant immune cell infiltration in these LUAD patients.

### Exploration of biological processes and pathways related to low ARID1A expression in LUAD cell lines using a multiomics method

**Fig. 2:**
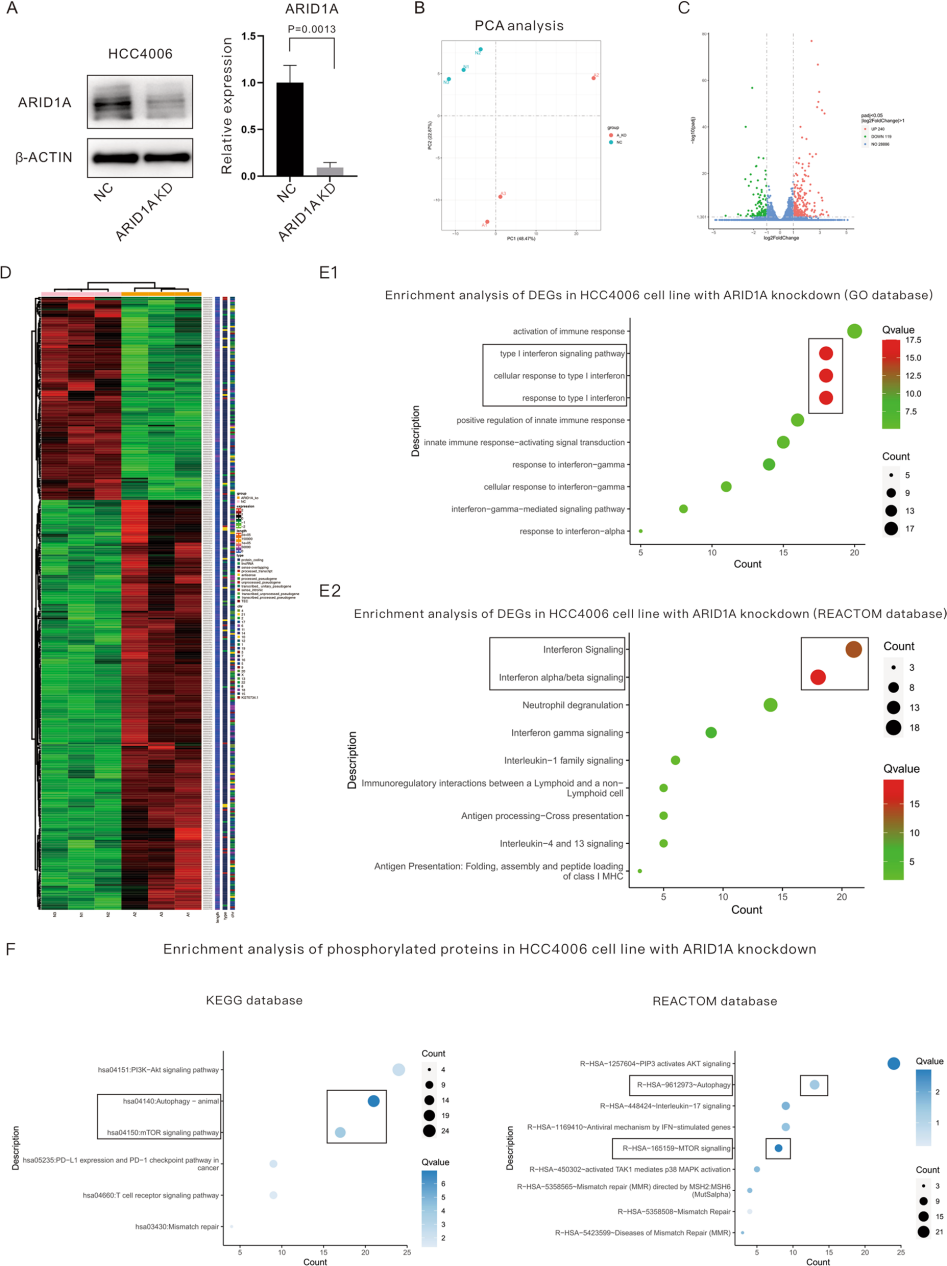
Exploration of biological processes and pathways related to low ARID1A expression in *EGFR*-mutant lung adenocarcinoma cell lines using a multiomics method. **A** Evaluation of ARID1A protein expression after construction of the ARID1A knockdown cell line. **B** PCA based on RNA samples for RNA-seq sequencing. **C** Volcano plot shows differentially expressed genes revealed by RNA-seq sequencing. **D** Heatmap for differentially expressed genes revealed by RNA-seq sequencing; **E1**, **E2** Enrichment analysis based on upregulated genes in ARID1A knockdown cells revealed by RNA-seq sequencing. **F** Enrichment analysis based on differentially expressed proteins revealed by mass spectrometry for upregulated phosphorylated proteins in ARID1A-knockdown cells

Bioinformatic analysis revealed the potential role of low ARID1A expression in LUAD immunotherapy. We thus further explored differences and underlying mechanisms induced by ARID1A expression changes using RNA-seq and MS for total proteins and phosphorylated proteins. The *EGFR*-mutant human LUAD cell line HCC4006 was implanted with sh-RNA for ARID1A and the NC vector after lentivirus infection. The expression level of ARID1A protein was significantly downregulated in ARID1A-KD compared with NC (*P* = 0.0013), as displayed in Fig. 2A. Principal component analysis (PCA) of RNA samples indicated that they can be separated well by PC1 and PC2 (Fig. 2B). The volcano plot in Fig. 2C demonstrates the DEGs between ARID1A-KD and NC cells based on RNA-seq. According to the DEGs revealed by RNA-seq (Fig. 2D), enrichment analysis was performed, as displayed in Fig. 2E1, E2, based on the GO database and REACTOM database, respectively. The DEGs were mainly enriched in interferon-related pathways, especially the type I IFN pathway, response to type I IFN, and activation of the interferon α/β signaling pathway. Other immune-related pathways, including the IFN γ signaling pathway and enhancement of antigen processing and immune response, were all significantly enriched in ARID1A-KD cells. To further clarify the specific signaling pathway altered in *EGFR*-mutant LUAD cells harboring ARID1A-KD, MS assays were performed to quantify phosphorylated proteins, and enrichment analysis was also performed based on DEPs revealed by MS, as shown in Fig. 2F. Interestingly, a significant change in phosphorylation level was detected in the PI3K/Akt/mTOR signaling pathway and the activity change of autophagy, which was revealed through enrichment analysis based on the KEGG database and REACTOM database. The results of RNA-seq based on RNA samples from the A549 cell line verified the findings above, as displayed in Additional file 2: Figure S1. The DEGs induced by ARID1A-KD in the A549 cell line are shown in Additional file 2: Figure S1A in a volcano plot format. ARID1A-KD significantly downregulated autophagy-related genes and upregulated IFN-related proteins (Additional file 2: Figure S1B). The enrichment analysis (Additional file 2: Figure S1C-S1D) based on DEGs revealed by RNA-seq in the A549 cell line also indicated that ARID1A-KD played an important role in influencing autophagy activity, anticancer immunity, type I IFN production and related pathways, according to the GO database and REACTOM database.

### ARID1A-KD activates the EGFR/PI3K/Akt/mTOR signaling pathway and inhibits autophagy in EGFR-mutant LUAD cells

**Fig. 3:**
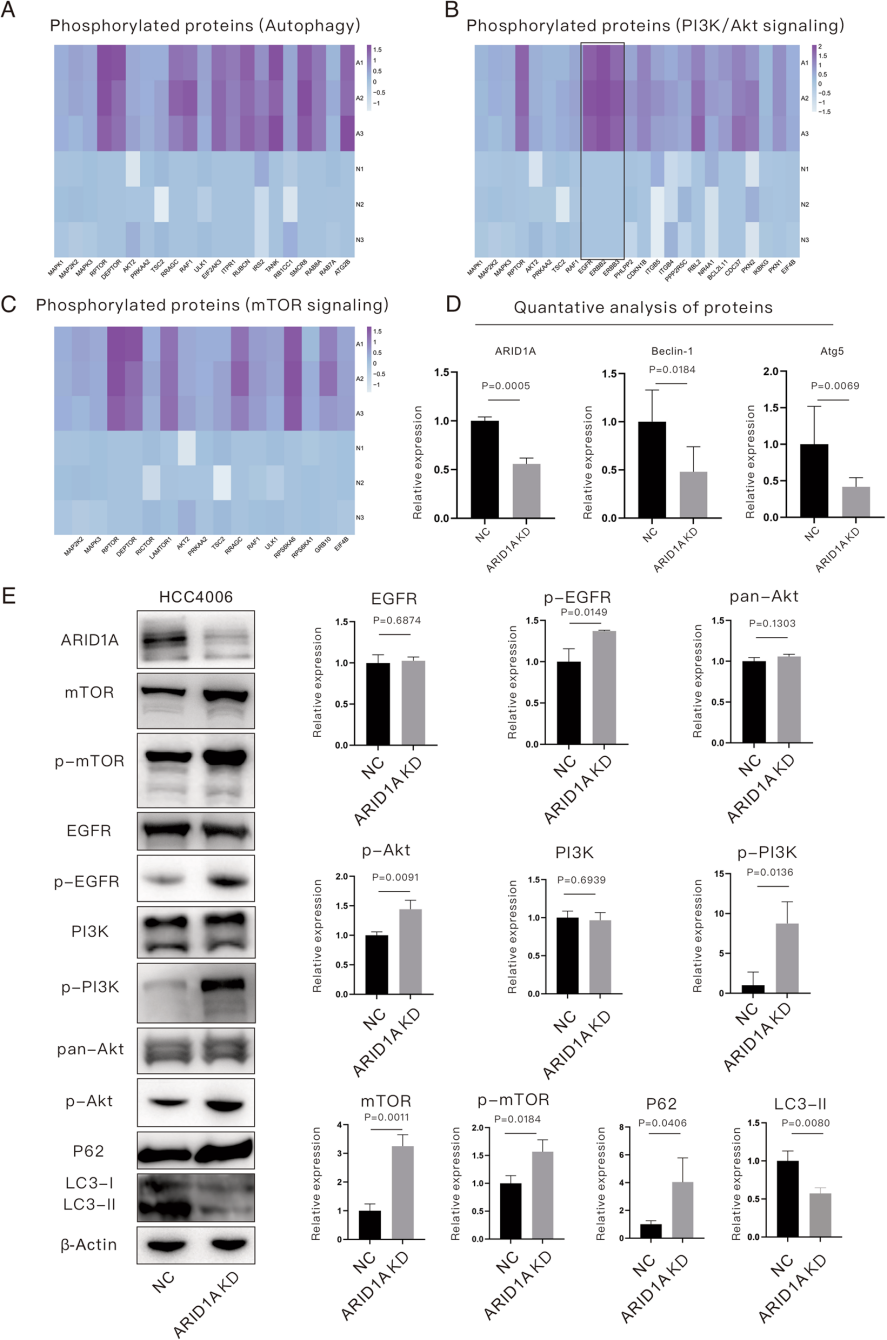
ARID1A-KD activates the EGFR/PI3K/Akt/mTOR signaling pathway and inhibits autophagy in *EGFR*-mutant lung adenocarcinoma cells. **A**–**C** Relative expression of phosphorylated proteins enriched in autophagy, the PI3K/Akt pathway and the mTOR pathway revealed by mass spectrometry. **D** Relative expression of autophagy-related biomarkers revealed by mass spectrometry. **E** Expression evaluation of targeted proteins revealed by western blotting

All of the phosphorylated proteins displayed in the heatmap in Fig. 3A–C were significantly upregulated in ARID1A-KD cells. The results demonstrated that phosphorylated RAPTOR in mTOR complex 1 (mTORC1) and RICTOR in mTOR complex 2 (mTORC2) were upregulated in ARID1A-KD cells. In addition, LAMTOR1 (an activator of mTOR) and DEPTOR (an inhibitor of mTOR) were activated in ARID1A-KD cells, creating confusion regarding the actual effects on mTOR signaling and autophagy. Therefore, subsequent MS for total proteins (Fig. 3D) and WB analysis for target proteins (Fig. 3E) were performed. ARID1A-KD significantly increased mTOR (*P* = 0.0011) and phosphorylated mTOR (p-mTOR, *P* = 0.0184) levels in HCC4006 cells (Fig. 3E). In ARID1A-KD cells, Beclin-1 (*P* = 0.0184) and autophagy-related 5 (Atg5, *P* = 0.0069) were downregulated (Fig. 3D), as was expression of LC3-II (*P* = 0.0080, Fig. 3E), but P62 (also called Sequestosome 1 [SQSTM1], *P* = 0.0406) was upregulated (Fig. 3E). All expression-level changes of the listed proteins suggest activation of the mTOR signaling pathway but inhibition of autophagy. MS indicated activation of the PI3K/Akt signaling pathway, which is an upstream pathway of mTOR, in ARID1A-KD cells (Fig. 3B). The members of the ErbB family, including EGFR, HER2 and ERBB3, were also activated in ARID1A-KD cells (Fig. 3B). WB analysis also confirmed increases in phosphorylated EGFR (p-EGFR, *P* = 0.0149), phosphorylated PI3K (p-PI3K, *P* = 0.0136) and phosphorylated Akt (p-Akt, *P* = 0.0091) proteins (Fig. 3E). Hence, ARID1A-KD activates the EGFR/PI3K/Akt/mTOR signaling pathway, which inhibits autophagy in *EGFR*-mutant LUAD cells.

### ARID1A-KD enhances production of type I interferon via the Rig-I-like receptor pathway in EGFR-mutant LUAD cells

**Fig. 4:**
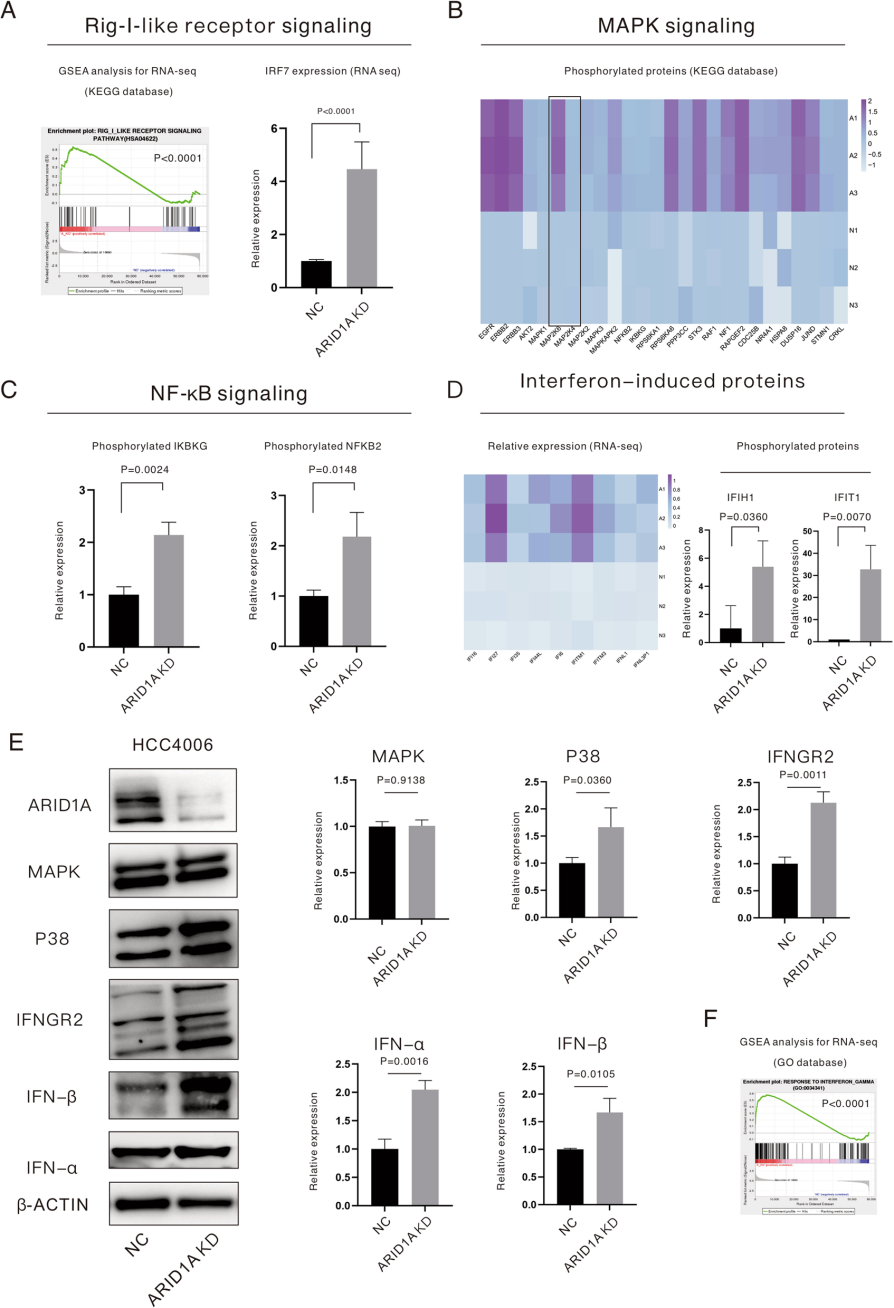
ARID1A knockdown enhances production of type I interferon via the Rig-I-like receptor pathway in *EGFR*-mutant lung adenocarcinoma cells. **A** GSEA revealed activation of the Rig-I-like receptor pathway; RNA-seq demonstrated upregulation of IRF7. **B** Relative expression of phosphorylated proteins enriched in the MAPK pathway revealed by mass spectrometry. **C** Relative expression of phosphorylated NF-κB pathway biomarkers revealed by mass spectrometry. **D** RNA-seq sequencing and mass spectrometry revealed upregulation of interferon-induced proteins. **E** Expression evaluation of targeted proteins revealed by western blotting. **F** GSEA revealed the enhancement of the response to interferon γ in ARID1A knockdown lung adenocarcinoma cells

GSEA based on the KEGG database suggested activation of the Rig-I-like receptor pathway (*P* < 0.0001). The three main downstream signaling pathways of the Rig-I-like receptor pathway, including the IRF7 pathway (Fig. 4A), MAPK pathway (Fig. 4B) and NF-κB pathway (Fig. 4C), were all stimulated in ARID1A-KD cells. The proteins listed in the heatmap in Fig. 4B, D were also significantly upregulated in ARID1A-KD cells. WB results for relative proteins are displayed in Fig. 4E. MS for total proteins showed significant upregulation of IRF7 (*P* < 0.0001, Fig. 4A). MS for phosphorylated proteins and WB suggested activation of the MAPK pathway and especially upregulation of phosphorylated MAP2K4 (Fig. 4B) as well as P38 MAPK (*P* = 0.0360, Fig. 4E). Phosphorylated IKBKG (*P* = 0.0024, Fig. 4C) and NF-κB2 (*P* = 0.0148, Fig. 4C) were upregulated in ARID1A-KD cells based on MS, which indicated attenuation of NF-κB inhibition and activation of the pathway, respectively. Given this evidence, the Rig-I-like receptor was activated, and one of the effects of this pathway is production of type I IFN. Through WB, expression of IFN-α (*P* = 0.0016) and IFN-β (*P* = 0.0105) was significantly upregulated in ARID1A-KD cells (Fig. 4E). RNA-seq and MS for phosphorylated proteins also revealed that IFN-induced proteins, such as IFI16, IFI27, IFI35, and IFI6, as well as phosphorylated IFIH1 (*P* = 0.0360) and IFIT1 (*P* = 0.0070), were obviously upregulated in ARID1A-KD cells, as displayed in Fig. 4D. Interestingly, we found that IFNGR2, which associates with IFNGR1 to form a receptor for the cytokine IFN-γ, was upregulated in ARID1A-KD cells (*P* = 0.0011, Fig. 4E). GSEA for DEGs based on the GO database suggested significantly improved response to IFN-γ in ARID1A-KD cells with *EGFR* mutation (*P* < 0.0001, Fig. 4F).

### Inhibition of autophagy in EGFR-mutant LUAD cells reverse type I IFN production, with an effect similar to ARID1A-KD

**Fig. 5:**
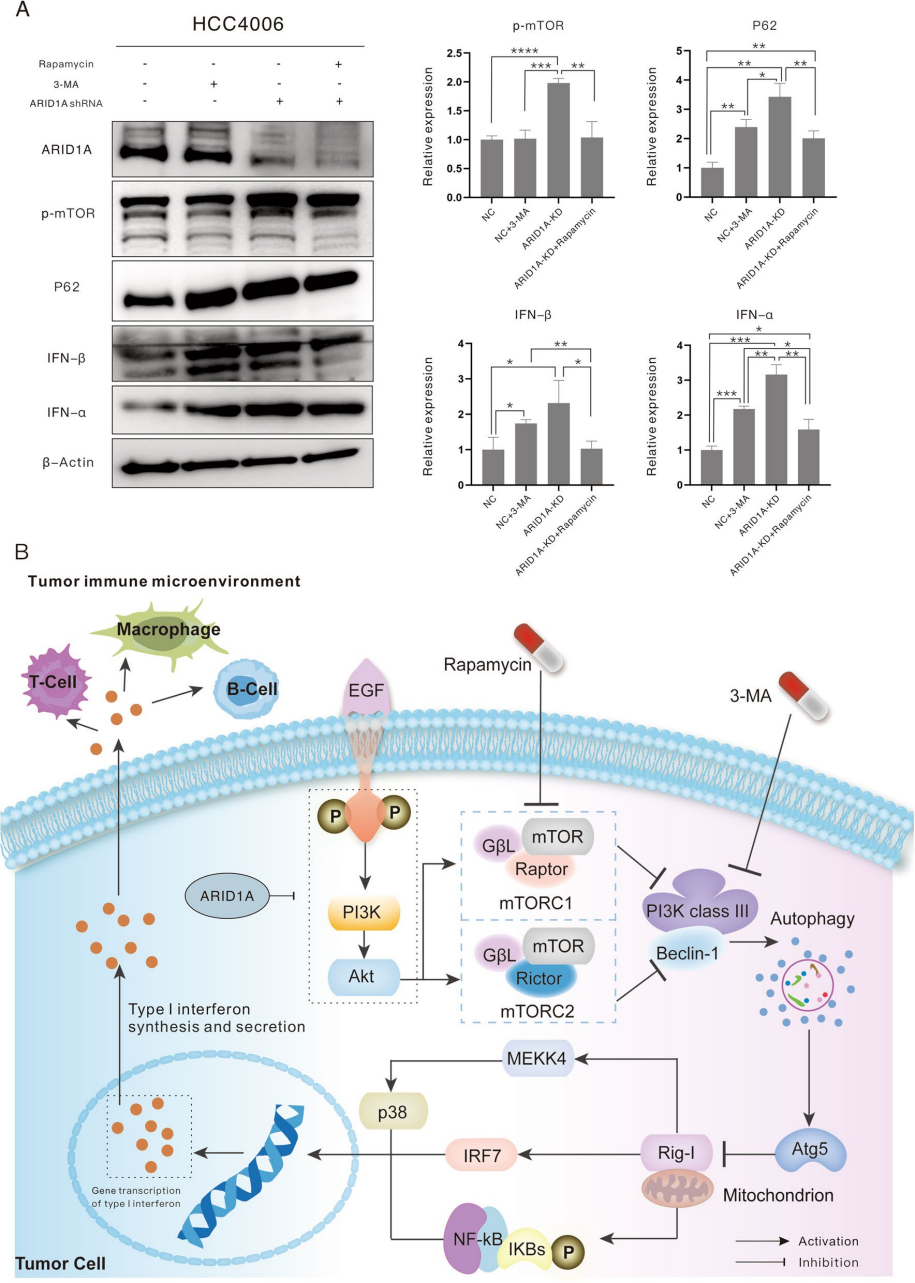
Inhibition of autophagy in *EGFR*-mutant lung adenocarcinoma cells reverses production of type I interferon, with a similar effect as ARID1A knockdown. **A** Expression evaluation of targeted proteins revealed by western blotting (3-MA: 2 mM; rapamycin: 100 µM). **B** Schematic diagram elucidates the mechanism of ARID1A knockdown in enhancing production of type I interferon in *EGFR*-mutant lung adenocarcinoma cells

According to the KEGG database (https://www.genome.jp/pathway/map04622), the process of autophagy, especially the protein Atg5, inhibits signaling from Rig-I to downstream pathways and type I IFN production. Therefore, we propose that inhibition of the autophagy process reverses type I IFN production in *EGFR*-mutant LUAD cells, similar to the effect of ARID1A-KD. Then, 2 mM 3-methyladenine (3-MA, SELLECK, S2767) and 100 µM rapamycin (SELLECK, S1039) were administered to NC and ARID1A-KD cells for 24 h [30]; total protein was harvested, and WB experiments were performed (Fig. 5A). Expression levels of p-mTOR and P62 were used to verify the drug effect on cells. Rapamycin inhibited the mTOR pathway in ARID1A-KD cells, downregulated expression of p-mTOR, and promoted autophagy, as indicated by downregulation of P62 expression. 3-MA inhibited the autophagy process and upregulated expression of P62 in NC cells. After treatment with the corresponding drug, production of type I IFN changed significantly. As displayed in Fig. 5A, inhibition of autophagy significantly elevated expression of IFN-α and IFN-β in NC cells, approaching the expression level in ARID1-KD cells. In contrast, autophagy promotion in ARID1A-KD cells obviously downregulated expression of IFN-α and IFN-β. In addition, after reversion of autophagy activity, production of IFN-α and IFN-β in NC cells was significantly higher than that in ARID1A-KD cells. In summary, autophagy activity in *EGFR*-mutant LUAD cells determines production of type I IFN.

To better elucidate the mechanism by which ARID1A-KD enhances production of type I IFN in *EGFR*-mutant LUAD cells, we plotted a schematic diagram, as depicted in Fig. 5B. Distinct activation of the EGFR/PI3K/Akt/mTOR pathway in ARID1A-KD LUAD cells harboring *EGFR* mutation inhibits autophagy activity and downregulates expression of autophagy-related proteins, such as Atg5. Moreover, downregulation of autophagy-related proteins attenuates inhibition of the Rig-I-like receptor pathway and downstream pathways, including the P38 MAPK, IRF7 and NF-κB pathways, and elevates type I IFN gene transcription, translation and secretion. Ultimately, type I IFN in the LUAD stroma increases immune cell infiltration, remodels the immune phenotype and reverses response to immunotherapy.

### EGFR-mutant LUAD patients with low ARID1A expression have enhanced immune filtration and a favorable response to anti-PD(L)1 therapy

**Fig. 6:**
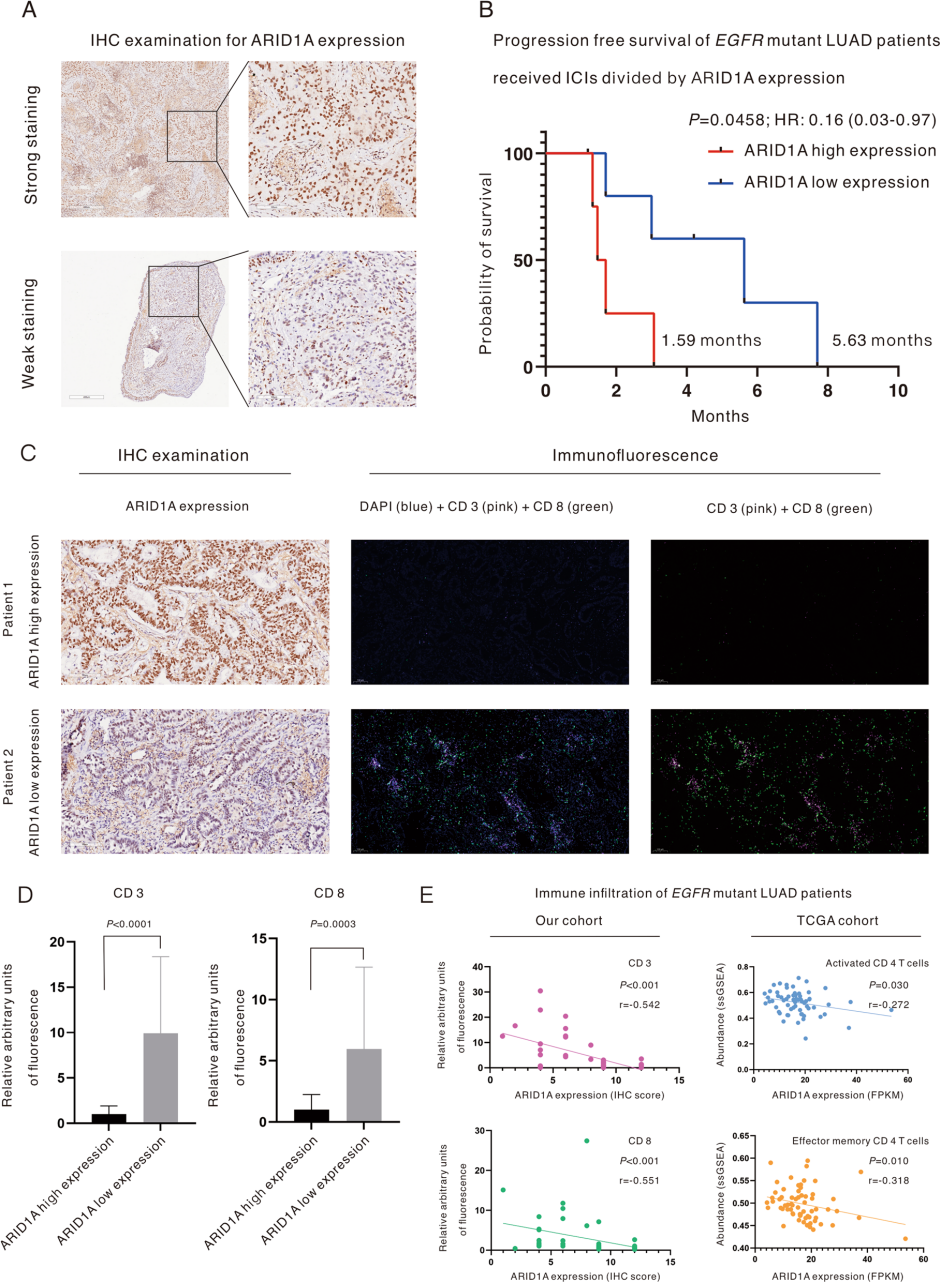
*EGFR*-mutant lung adenocarcinoma patients with low ARID1A expression have enhanced immune cell filtration and a favorable response to anti-PD(L)1 therapy. **A** Representative images for IHC examination of ARID1A protein. **B** Survival analysis of progression-free survival of *EGFR-*mutant lung adenocarcinoma patients who received immune checkpoint inhibitors divided by ARID1A expression. **C** Representative images for the immunofluorescence assay to evaluate immune cell infiltration in *EGFR*-mutant lung adenocarcinoma patients. **D** Low ARID1A expression was associated with higher arbitrary units of CD3 and CD8, as revealed by the immunofluorescence assay. **E** ARID1A correlates negatively with immune cell infiltration in *EGFR*-mutant lung adenocarcinoma patients

The evidence above indicates the mechanisms related to ARID1A-KD through which a better response to ICIs occurs, and further verification was performed using *EGFR*-mutant LUAD cohorts from our cancer center and TCGA. In total, 10 *EGFR*-mutant LUAD patients who received ICIs combined with chemotherapy and/or antiangiogenic therapy were enrolled for survival analysis based on ARID1A expression, and representative images of IHC for ARID1A are displayed in Fig. 6A. *EGFR*-mutant LUAD patients with low ARID1A expression had better progression-free survival (PFS) than those with high ARID1A expression (5.63 months versus 1.59 months, *P* = 0.0458), and the hazard ratio (HR) was 0.16 (95% confidential interval [CI]: 0.03–0.97) (Fig. 6B). The ARID1A low expression group had a higher disease control rate (DCR) than the high expression group (80% versus 25%) in the best response to ICI treatment. Furthermore, we estimated immune cell infiltration, mainly including IFA signals (AU) of CD3 and CD8, in the enrolled *EGFR*-mutant LUAD patients (n = 49). Low ARID1A expression was associated with more abundant AU of both CD3 and CD8 (Fig. 6C). Quantitatively, as shown in Fig. 6D, *EGFR*-mutant LUAD patients with low ARID1A expression had higher AU of CD3 (*P* < 0.0001) and CD8 (*P* = 0.0003) compared with the ARID1A high expression group. The linear correlation between ARID1A expression and immune cell infiltration was then detected in *EGFR*-mutant LUAD patients, as illustrated in Fig. 6E. ARID1A expression correlated negatively with CD3-positive T cells (*P* < 0.0010, r = − 0.542) and CD8-positive T cells (*P* < 0.0010, r = − 0.551) in our cohort of patients and with activated CD4-positive T cells (*P* = 0.0300, r = − 0.272) and effector memory CD4-positive T cells (*P* = 0.0100, r = − 0.318) in the cohort of patients from TCGA.

## Discussion

*EGFR* mutations in NSCLC patients are associated with poor response to ICI treatment, and only approximately 20% of NSCLC patients harboring *EGFR* mutation benefit from immunotherapy [12]. Overall, novel biomarkers or therapeutics are urgently needed to predict prognosis or enhance the efficacy of immunotherapy in NSCLC patients harboring *EGFR* mutation, especially patients with LUAD, which accounts for approximately 40–50% of all NSCLC cases. Previous research has revealed that *ARID1A* mutation or ARID1A expression loss contributes to the dMMR subtype, higher PD-L1 expression and remodeling of the TIME, which leads to enhanced sensitivity to ICIs in ovarian cancer [31] and gastric cancer [32]. In addition, it was reported that ARID1A depletion limits acquisition of exhaustion-associated chromatin accessibility and leads to improved anticancer immunity [33]. The results of the current study offer clear support for the role of low ARID1A expression as a positive biomarker for ICI treatment in LUAD patients with or without *EGFR* mutation. First, ARID1A expression correlates negatively with scores for immune evaluation, including the ESTIMATE score, immune score and IPS score, for PD-1/PD-L1 blockade in LUAD patients with or without *EGFR* mutation, which predicts a better response to ICI treatment. Second, ARID1A expression correlates negatively with the abundance of immune cell infiltration, especially infiltration of T lymphocytes, as represented by the enriched IFA signal of CD3 (total T lymphocytes) and CD8 (CTL) in our cohort and enriched CD4-positive T cells in the cohort from TCGA for *EGFR*-mutant LUAD patients. The enhanced immune infiltration, especially CD8 positive CTLs, would improve the response to anti-cancer immunotherapy. In addition, survival analysis confirmed ARID1A deficiency to be associated with longer PFS in *EGFR*-mutant LUAD patients (our cohort) and longer OS in pancancer patients (cBioPortal). All this evidence suggests the essential role of ARID1A deficiency in enhancing the efficacy of ICIs in *EGFR-*mutant LUAD patients, but the underlying mechanisms remain to be further explored. As a result, we performed subsequent experiments.

Mechanistically, roles for autophagy in remodeling the TIME and influencing immunotherapy are becoming increasingly evident. Autophagy mediates major histocompatibility complex (MHC) class I degradation, which reduces the immunogenicity of tumor cells and contributes to immune suppression. In addition, autophagy blocks CTL-mediated and NK-cell-mediated tumor killing and inhibits chemokine CCL5 expression, which is required for recruitment of NK-cell migration to the TIME and ultimately results in immune escape in tumor cells [34]. Another study found that 3-MA, an autophagy inhibitor, enhances activation of CD8-positive T cells in tumor tissue [35], confirming the negative role of autophagy in regulating anticancer immunity. Our results suggest that ARID1A-KD activates the EGFR/PI3K/Akt/mTOR pathway and inhibits the level of autophagy in *EGFR*-mutant LUAD cells. ARD1A-KD upregulates expression of both the mTOR protein and its phosphorylated form, which strongly reveals that ARID1A-KD may serve as a biomarker of autophagy inhibition in *EGFR-*mutant LUAD cells. The latest research sheds new light on type I IFN in enhancing response to ICIs in LUAD [36]. For example, upregulation of type I IFN promotes accumulation of CD8-positive T cells to improve anticancer immunity. In the current study, we propose that ARID1A-KD significantly strengthens endogenous production of type I IFN, mainly including IFN-α and IFN-β, in *EGFR-*mutant LUAD cells via activation of the Rig-I-like receptor pathway and its downstream pathways, including the IRF7, NF-κB and MAPK pathways [37].

Two mechanisms, including inhibition of autophagy and promotion of type I IFN production, were detected and confirmed in ARID1A-KD *EGFR*-mutant LUAD cells, and we sought to explore whether there is a connection between these two mechanisms in *EGFR*-mutant LUAD cells. A previous review suggested that production of type I IFN is stimulated in autophagy-deficient cancer cells [33]. For *EGFR*-mutant LUAD cells, we found that type I IFN production was inhibited by autophagy and that knockdown of ARID1A-KD could reverse production of type I IFN via inhibition of autophagy. In addition, autophagy inhibitors, such as 3-MA, upregulated production of type I IFN in *EGFR*-mutant LUAD cells, with a similar effect as ARID1A-KD. Conversely, autophagy promoters, such as rapamycin (mTOR inhibitor), attenuated this enhanced production of type I IFN in ARID1A-KD *EGFR*-mutant LUAD cells. Together, the present findings confirm that ARID1A-KD reverses the response to anti-PD(L)1 therapy in *EGFR*-mutant LUAD by enhancing autophagy-inhibited type I IFN production. Another novel finding is that autophagy inhibitors can be used as additional therapeutics for *EGFR*-mutant LUAD patients under ICI treatment. The same mechanisms were also found for the *EGFR*-wild-type cell line, which suggests that inhibition of autophagy is a potential treatment option for immunotherapy in LUAD patients with wild-type *EGFR*. Admittedly, several limitations exist in our study, including a lack of in vivo verification and the small sample size of *EGFR*-mutant LUAD patients who received ICI treatment. An expanded study is needed to further confirm the role of ARID1A in cancer immunotherapy.

## Conclusion

Low ARID1A expression is associated with infiltrating immune cell accumulation and a better response to ICI treatment in *EGFR*-mutant LUAD patients. Mechanistically, ARID1A-KD reverses the response to anti-PD(L)1 therapy in *EGFR*-mutant LUAD by enhancing autophagy-inhibited type I interferon production. Furthermore, autophagy inhibitors may be used as additional therapeutics for *EGFR*-mutant LUAD patients receiving ICI treatment.

## Supplementary Information

**Additional file 1. Table S1:** Antibodies and lentivirus sequences.

**Additional file 2. Figure S1: **Results of RNA-seq of the A549 cell line. A. The volcano plot of differentially expressed genes revealed by RNA-seq sequencing. B. Heatmap for autophagy- or interferon-related gene expression. C. Enrichment analysis for differentially expressed genes based on the GO database. D. Enrichment analysis for differentially expressed genes based on the REACTOM database.

**Additional file 3.** Information for the STR Cell ID assay.

## Data Availability

All data and materials are mentioned in this article and can be requested by email.

## Abbreviations

LUAD: Lung adenocarcinoma
NSCLC: Non-small cell lung cancer
EGFR: Epidermal growth factor receptor
TKIs: Tyrosine kinase inhibitors
ALK: Anaplastic lymphoma kinase
ICIs: Immune checkpoint inhibitors
OS: Overall survival
anti-PD(L)1: Anti-programmed cell death (ligand) 1
SWI/SNF: Switch/sucrose nonfermenting
MMR: Mismatch repair
TMB: Tumor mutation burden
TILs: Tumor-infiltrating lymphocytes
ARID1A-KD: ARID1A knockdown
IFN: Interferon
TIME: Tumor immune microenvironment
TCGA: The Cancer Genome Atlas
ssGSEA: Single-sample gene set enrichment analysis
DEGs: Differentially expressed genes
GSEA: Gene set enrichment analysis
H&E: Hematoxylin and eosin
IHC: Immunohistochemical
RECIST 1.1: Response evaluation criteria in solid tumors 1.1
AJCC: American Joint Committee on Cancer
FBS: Fetal bovine serum
CTLs: Cytotoxic lymphocytes
DEPs: Differentially expressed
BIC: Bioinfo Intelligent Cloud
PFS: Progression-free survival
NK: Natural killer
mTORC1: MTOR complex 1
mTORC2: MTOR complex 2
HR: Hazard ratio
