# Supplementary material for: ARID1A deficiency reverses the response to anti-PD(L)1 therapy in EGFR-mutant lung adenocarcinoma by enhancing autophagy-inhibited type I interferon production
Source: Cell Commun Signal. 2022 Oct 13;20:156. doi: 10.1186/s12964-022-00958-5 (PMC9558404; doi:10.1186/s12964-022-00958-5)
Supplement: Supplementary file 2 — Additional file 1. Table S1: Antibodies and lentivirus sequences. [file 12964_2022_958_MOESM2_ESM.doc]

Table S1. Antibodies and lentivirus sequences

| **Antibodies** | | | |
| --- | --- | --- | --- |
| **Name** | **Manufacturer** | **Number** | **Dilution rate** |
| ARID1A | Abcam | ab182560 | 1:500 (IHC); 1:1000 (WB) |
| mTOR | Cell Signaling Technology | 2983 | 1:1000 |
| phosphorylated-mTOR | Cell Signaling Technology | 5536 | 1:1000 |
| EGFR | Cell Signaling Technology | 4267 | 1:1000 |
| phosphorylated-EGFR | Cell Signaling Technology | 3777 | 1:1000 |
| PI3K | Cell Signaling Technology | 4249 | 1:1000 |
| phosphorylated-PI3K | Cell Signaling Technology | 17366 | 1:1000 |
| P62 (SQSTM1) | KleanAB | P113161 | 1:1000 |
| MAP1LC3B | KleanAB | P111215 | 1:1000 |
| pan-AKT | Cell Signaling Technology | 4685 | 1:1000 |
| phosphorylated-AKT | Cell Signaling Technology | 4060 | 1:2000 |
| STAT3 | Cell Signaling Technology | 12640 | 1:1000 |
| phosphorylated-STAT3 | Cell Signaling Technology | 9145 | 1:2000 |
| MAPK | Cell Signaling Technology | 4695 | 1:2000 |
| P38 MAPK | Cell Signaling Technology | 51255 | 1:1000 |
| IFNGR2 | KleanAB | P102799 | 1:500 |
| IFNA1/A13 | KleanAB | P112773 | 1:500 |
| IFNB1 | KleanAB | P110960 | 1:1000 |
| β-actin | Sigma-Aldrich | A3854 | 1:5000 |
| CD3 (IFA) | Servicebio | GB13440 | 1:1000 |
| CD8 (IFA) | Servicebio | GB13068-2 | 1:500 |
| Secondary antibody | Servicebio | GB23303 | 1:500 |
| Secondary antibody | Servicebio | GB23301 | 1:500 |
| Secondary antibody | jackson | 594-labeled goat anti-rabbit | 1:400 |
| **Lentivirus sequences (5’-3’)** | | | |
| sh-ARID1A | GTTGATGAACTCATTGGTT | | |
| Vector control | TTCTCCGAACGTGTCACGT | | |
