# Supplementary figures and images for: ARID1A deficiency reverses the response to anti-PD(L)1 therapy in EGFR-mutant lung adenocarcinoma by enhancing autophagy-inhibited type I interferon production
Source: Cell Commun Signal. 2022 Oct 13;20:156. doi: 10.1186/s12964-022-00958-5 (PMC9558404; doi:10.1186/s12964-022-00958-5)

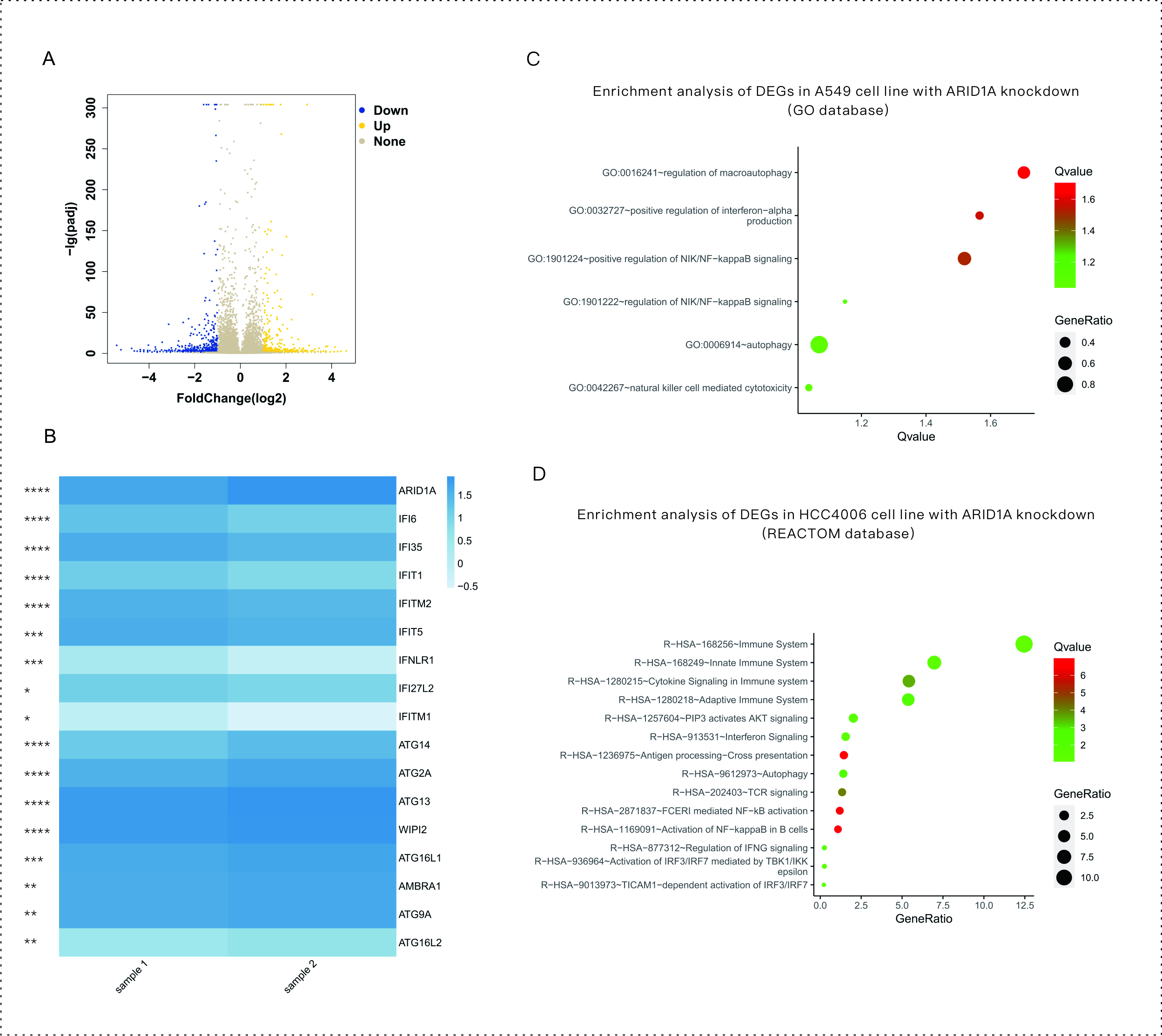

Supplement: Supplementary file 3 — Additional file 2. Figure S1: Results of RNA-seq of the A549 cell line. A. The volcano plot of differentially expressed genes revealed by RNA-seq sequencing. B. Heatmap for autophagy- or interferon-related gene expression. C. Enrichment analysis for differentially expressed genes based on the GO database. D. Enrichment analysis for differentially expressed genes based on the REACTOM database. [file 12964_2022_958_MOESM3_ESM.jpg]
